# The relationship between the evolution of microRNA targets and the length of their UTRs

**DOI:** 10.1186/1471-2164-10-431

**Published:** 2009-09-14

**Authors:** Chao Cheng, Nitin Bhardwaj, Mark Gerstein

**Affiliations:** 1 Program in Computational Biology and Bioinformatics, Yale University, New Haven, CT 06520, USA; 2 Department of Molecular Biophysics and Biochemistry, Yale University, New Haven, CT 06520, USA; 3 Department of Computer Science, Yale University, New Haven, CT 06520, USA

## Abstract

**Background:**

MicroRNAs (miRNAs) are endogenous small RNA molecules that modulate the gene expression at the post-transcription levels in many eukaryotic cells. Their widespread and important role in animals is gauged by estimates that ~25% of all genes are miRNA targets.

**Results:**

We perform a systematic investigation of the relationship between miRNA regulation and their targets' evolution in two mammals: human and mouse. We find genes with longer 3' UTRs are regulated by more distinct types of miRNAs. These genes correspondingly tend to have slower evolutionary rates at the protein level. Housekeeping genes are another class of genes that evolve slowly. However, they have a distinctly different type of regulation, with shorter 3'UTRs to avoid miRNA targeting.

**Conclusion:**

Our analysis suggests a two-way evolutionary mechanism for miRNA targets on the basis of their cellular roles and the length of their 3' UTRs. Functionally critical genes that are spatially or temporally expressed are stringently regulated by miRNAs. While housekeeping genes, however conserved, are selected to have shorter 3'UTRs to avoid miRNA regulation.

## Background

Regulation of gene expression at the transcriptional level plays a central role in governing all cellular activities. However, the significance of gene regulation at the post-transcriptional level has gained a lot of interest and popularity over the last decade. MicroRNAs (miRNAs) are one of the two regulators, along with siRNA (short interfering RNAs), that have emerged as important players in post-translation regulation and mRNA decay. miRNAs are endogenously expressed small RNAs that regulate gene expression at the post-transcriptional level [1,2]. To repress expression of mRNAs, miRNAs recognize target sites in their 3' un-translated region (3'UTR) via base pairing, leading to their degradation or inhibition of their translation [3-5]. Considering the critical roles of miRNAs in gene expression regulation [6-8], it would be interesting and insightful to investigate the regulatory effect of miRNAs from an evolutionary perspective.

Intuitively, for functionally important proteins that contribute significantly to individual fitness, selection pressure may exhibit its effect in two aspects. On one hand, non-synonymous mutations that lead to slightly deleterious substitutions accumulate slowly in these proteins [9]. On the other hand, expression of genes encoding these proteins is subjected to delicate but robust regulation at the transcriptional and post-transcriptional levels. Therefore, genes under more stringent regulation by miRNAs are expected to evolve more slowly at the protein level. In this study, we investigated the relationship between miRNA regulation and protein evolutionary rate in two mammals: human and mouse. For these two species, a large number of miRNAs have been identified that enables a systematic analysis with statistically significant conclusions.

## Results and Discussion

### Protein evolutionary rates are negatively correlated with the number of regulatory miRNAs

First, we calculated the number of distinct regulatory miRNAs for each human and mouse gene based on the predicted miRNA binding sites by the PITA algorithm [10]. We chose PITA for miRNA target prediction because it has been shown to achieve high prediction accuracy, and more importantly, it takes advantage of the target accessibility but not conservation information (used by most of the other methods) to reduce false positives [10]. Such an omission is important for this study because we find that the conservation at 3'UTR is correlated with the conservation at the coding regions as well as the protein evolutionary rate, and therefore it may complicate our analysis. Specifically, we calculated the average conservation score of 3'UTR and coding regions for all human mRNAs according to the sequence alignment of 17 vertebrate species [11]. The results indicate that the conservation score at 3'UTR is positively correlated with that at the coding region (ρ = 0.55) and negatively correlated with the human protein evolutionary rate Ka/Ks against mouse (ρ = -0.43). The second reason for using accessibility over conservation information is that previous experiments have shown that, in addition to conserved miRNAs target sites, non-conserved sites are also functional and mediate repression [12,13].

**Figure 1:**
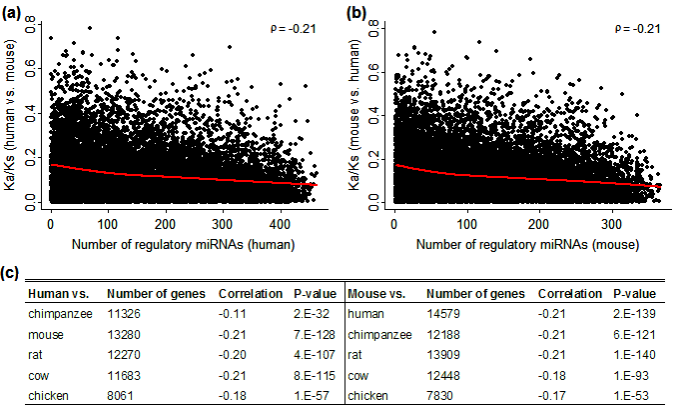
**The relation between the number of regulatory miRNAs and the protein evolutionary rate**. (a, b) The evolutionary rates of proteins in human (a) and mouse (b) are estimated as the Ka/Ks ratios using the other organism as reference. The number of regulatory miRNAs is calculated by counting miRNAs that have at least one target site within the 3'UTR of a gene. The red lines show the smoothed relation between the two quantities estimated by LOESS method. *ρ *indicates Spearman correlation coefficient. (c) Correlation between the number of regulatory miRNAs and protein evolution rate using various species as references. miRNA binding sites are based on PITA predictions.

Next, using the homologous pairs between mouse and human from HomoloGene [14], we obtained the evolutionary rates of human and mouse proteins. Evolutionary rate for protein is defined as the Ka/Ks ratio, where Ka and Ks are the rates of non-synonymous and synonymous substitutions, respectively. Finally, for those genes for which we had both miRNA target site predictions and evolutionary information, we calculated the correlation between the number of distinct regulatory miRNAs and the protein's evolutionary rates. Our results indicate a significant negative correlation between the two in both human (Spearman correlation coefficient, ρ = -0.21, P = 7E-128 when the Ka/Ks ratios are obtained by aligning against homologous proteins in mouse; Figure 1a) and mouse (ρ = -0.21, P = 2E-139 when the Ka/Ks ratios are obtained using human as the reference; Figure 1b), suggesting that genes regulated by more distinct miRNAs at the 3'UTR regions tend to have slower evolutionary rates. Similar trends were obtained when other species ranging from chicken to human are used as references to obtain the evolutionary rates (Figure 1c). We also performed the same analysis using two other miRNA target site prediction methods: miRanda and TargetScan [15-19]. For the reasons stated above, we did not impose conservation filtering but kept all the potential miRNA target sites in our analysis. Again, our results indicate a significant negative correlation between the number of regulatory miRNAs and protein evolutionary rate in both human (ρ = -0.17, P = 5E-70 when the Ka/Ks ratios are obtained using mouse as the reference with miRanda; ρ = -0.187, P = 2E-80 with TargetScan) and mouse (ρ = -0.11, P = 2E-15 when the Ka/Ks ratios are obtained using human as the reference; see Additional file 1: Figure S1). This demonstrates that our results are robust to the way protein evolutionary rates are obtained and to the miRNA target site prediction method (see Additional file 2: Table S1 and Additional file 3, 4, 5 &6: Figure S2-S5).

### The negative correlation is independent of the expression intensities of miRNA targets

It can be argued that the intensity of gene expression, which relates inversely to the rate of protein sequence evolution [20], could be the underlying cause of the negative correlation between number of regulatory miRNAs and evolutionary rate. To rule out this possibility, we calculated the average expression intensities of human genes in 79 tissues [21]. Our results indicate a negative correlation between average expression level of human genes and their evolutionary rates (ρ = -0.18, P = 7E-70 when the Ka/Ks ratios are obtained using mouse as the reference). However, there is no significant correlation between the number of regulatory miRNAs and gene expression intensities (ρ = -0.016, P = 0.12). Therefore, the negative correlation between the number of regulatory miRNAs and the protein's evolutionary rate is unlikely to be mediated by gene expression intensities. This argument is further validated by parametric (-0.20, P = 1E-85) and non-parametric (-0.20, P = 2E-86) partial correlation coefficients between the number of regulatory miRNAs and evolutionary rate with the expression intensity being held constant [22].

### Genes with longer 3'UTR tend to evolve at slower rates

**Figure 2:**
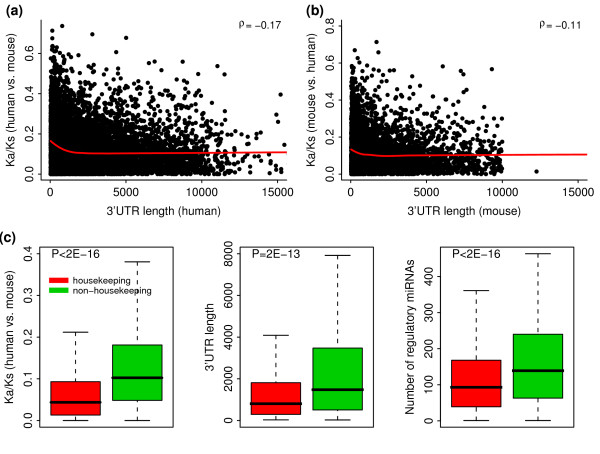
**The relation between protein evolutionary rate and 3'UTR length of mRNAs**. (a, b) Global inverse relationship between protein evolutionary rate and 3'UTR length for human (a) and mouse (b) mRNAs. (c) Difference between housekeeping (red) and non-housekeeping (green) human genes in evolutionary rate (Left), 3'UTR length (Middle) and the number of regulatory miRNAs (Right). The P-values are calculated using the Wilcoxon rank sum test.

In general, genes with longer 3'UTRs are likely to be regulated by more miRNAs. We therefore examine the correlation between 3'UTR length and evolutionary rate. As expected, we find that there is a negative correlation between these two in both human (ρ = -0.17, P = 6E-72 when the Ka/Ks ratios are obtained using mouse as the reference, Figure 2a) and mouse (ρ = -0.11, P = 7E-17 when the Ka/Ks ratios are obtained using human as the reference, Figure 2b). Similar results were obtained using miRNA targets predicted by the miRanda and TargetsScan algorithms (see Additional file 7: Table S2).

Previous studies, however, have shown that housekeeping genes are likely to have shorter 3'UTRs to avoid miRNA regulation, suggesting that they may have a different scenario in terms of miRNA regulation and protein evolution [23,24]. So, we compared the human housekeeping [23] with non-housekeeping genes and found that housekeeping genes are more likely to have slower evolutionary rates, shorter 3'UTRs and less number of regulatory miRNAs (Figure 2c). Interestingly, a recent study demonstrated the increased relative expression of the mRNA isoforms with shortened 3'UTR and fewer miRNA target sites in proliferating cells, suggesting that modulating 3'UTR length through alternative splicing is likely to be a biological mechanism to adjust miRNA regulation [25]. However, it should be noted here that while miRNAs overall target longer UTRs, the number of target sites does not simply scale with the length; rather, target sites are preferentially found towards the end of the UTRs [26-28].

### Correlation between number of miRNAs and evolutionary rate is beyond the length of the 3'UTR region

We wanted to examine if the negative correlation between evolutionary rate and number of regulating miRNAs goes beyond the length of the 3'UTR region. So, we integrated a control for the length bias and found that there is no significant correlation between the density of miRNA binding and the protein evolutionary rate (ω), which may indicate that change of the number of regulatory miRNAs for genes is mainly achieved by change of its 3'UTR length and requires no change of binding site density. However, the correlation between the number of miRNAs (N) and protein evolutionary rate (ω) cannot be fully explained by 3'UTR length (L) as indicated by partial correlation coefficients: for human ρ(ω, N | L) = -0.172 (P = 1E-60) when PITA is used; ρ(ω, N | L) = -0.151 (P = 6E-53) when TargetScan is used. On the other hand, the correlation between ω and L is largely explained by N: ρ(ω, L | N) = -0.044 (P = 3E-5) when PITA is used and ρ(ω, L | N) = -0.048 (P = 1E-6) when TargetScan is used. These results suggest that anti-correlation between evolutionary rates and number of miRNAs is not mediated by the 3'UTR length.

We further show that the correlation between protein evolution and the number of miRNA binding sites is mediated by *functional *miRNA binding sites in the 3'UTR region. We do so by generating shuffled miRNAs with permuted nucleotide sequences while keeping the length and base composition unchanged in human. We predicted targets of miRNAs in a similar way as TargetScan - searching for the presence of 8 mer (exact match to positions 2-8 of the mature miRNA followed by an 'A'), 7 mer-m8 (exact match to positions 2-8 of the mature miRNA) and 7 mer-1A (exact match to positions 2-7 of the mature miRNA followed by an 'A') sites that match the seed region of each miRNA. Our results indicate a significant correlation between the number of shuffled miRNAs for a gene and the protein evolution rate (computed as Ka/Ks against mouse), which is expected due to the strong correlation between 3'UTR length and the number of shuffled miRNA binding sites. However, after taking into account the 3'UTR length, the correlation between them is abolished as shown by the partial correlation ρ(ω, N | L) = 0.014 (P > 0.05) indicating that correlation between evolutionary rate and the number of shuffled miRNA binding sites can be fully explained by 3'UTR length.

### Correlation of genetic features with evolutionary rate

We also determined the correlation of protein evolutionary rate (Ka/Ks ratio) with 5 gene features: 5'UTR length, CDS length, cDNA length, first exon length and first intron length. All these features have negative correlation with Ka/Ks ratios showing that more conserved proteins demonstrate higher values of the above features. But a more careful investigation indicated that this anti-correlation is due to the correlation between these features with 3'UTR length. There is only small (with the exception of first intron length) yet moderately significant correlation between them and the protein evolutionary rate after taking 3'UTR length into account as indicated by their partial correlations: -0.058 for 5'UTR, -0.026 for CDS, -0.032 for cDNA, -0.033 for first exon and -0.10 for first intron lengths, respectively. On the other hand, after taking into account these features, the partial correlation between 3'UTR length and Ka/Ks ratio is still considerable: -0.16, -0.16, -0.10, -0.16 and -0.15 when the 5'UTR, CDS, cDNA, first exon and first intron lengths are held constant, respectively. Therefore, it seems that more conserved proteins tend to have longer 3'UTRs and first introns. The correlation between first intron length and protein conservation is interesting and indicates that factors other than miRNA regulation also shape protein evolution.

## Conclusion

To understand how natural selection has shaped the evolution of miRNAs and their target genes, some past exploratory studies have been performed but they all have focused on the evolution of miRNAs or their target sites in 3'UTR region [12,13,24,29,30]. In this novel study, we investigated how miRNA regulation is correlated with the evolution of proteins in human and mouse. Our results suggest that a two-way strategy has been implemented in mammals to achieve stringent regulation of genes at post-transcriptional level by miRNAs. First, functionally critical genes that are spatially or temporally expressed (non-housekeeping genes) are stringently regulated by miRNAs. For robust regulation of these genes, longer 3'UTRs are preferred so that more target sites of distinct regulatory miRNAs can be included. Secondly, housekeeping genes, however conserved, are selected to have shorter 3'UTRs to avoid miRNA regulation.

## Methods

miRNA target prediction data by PITA [10] was downloaded from . The data contained binding information for 475 human and 375 mouse miRNAs at the 3'UTR regions of mRNAs for the two species. To predict miRNA targets using the miRanda method [16], we downloaded the human and mouse 3'UTR sequences from the PolyA Cleavage Site and 3'-UTR Database [31] which is available at . To ensure prediction accuracy, only 3'UTR sequences labeled with "very high confidence" in the database were included in our analysis. An mRNA could correspond to multiple 3'UTR sequences due to alternative splicing and in such cases the longest sequence was used.

The evolutionary rate for human and mouse proteins, measured by Ka/Ks ratio, were calculated based on data from the HomoloGene database [14] available at . The phastCons score, which is a measure of evolutionary conservation in 17 vertebrates, was downloaded from the UCSC Genome Browser at . To calculate the conservation score for the 3'UTR or coding region of a specific mRNA, the phastCons scores of all nucleotides within it were averaged. Human housekeeping and non-housekeeping genes are categorized based previous work by Eisenberg et al. [23]. All calculations and analyses are performed using the R platform.

## Authors' contributions

CC conceived of the study, carried out the data analysis and helped in drafting the manuscript. NB participated in the design of the study and helped in drafting the manuscript. MG conceived of the study, and participated in its design and coordination and helped to draft the manuscript. All authors read and approved the final manuscript.

## Supplementary Material

Additional file 1 **Figure S1: The relation between number of regulatory miRNAs predicted by miRanda method and protein evolutionary rate in human (A) and mouse (B)**. Both Pearson correlation coefficient (PCC) and Spearman correlation coefficient (SCC) are shown. Click here for file

Additional file 2 **Table S1: correlation between the number of regulatory miRNAs predicted by miRanda method and protein evolutionary rate using various species as references**. Correlation shown in the table is Spearman correlation coefficient. Click here for file

Additional file 3 **Figure S2: The relation between number of regulatory human miRNAs predicetd by miRanda method and protein evolutionary rate using chimpanzee (A), rat (B), cow (C), and chicken (D) as reference, respectively**. The number of regulatory miRNAs is calculated by counting miRNAs that have at least one target site within the 3'UTR of a gene. The red lines show the smoothed relation between evolutionary rate and number of regulatory miRNAs estimated by LOESS method. PCC and SCC represent Pearson correlation coefficient and Spearman correlation coefficient, respectively. Click here for file

Additional file 4 **Figure S3: The relation between number of regulatory mouse miRNAs prediceted by miRanda method and protein evolutionary rate using human (A), chimpanzee (B), cow (C), and chicken (D) as reference, respectively**. Figure legends are similar to those in Additional file 3: Figure S2. Click here for file

Additional file 5 **Figure S4: The relation between number of regulatory human miRNAs predicted by PITA method and protein evolutionary rate using chimpanzee (A), rat (B), cow (C), and chicken (D) as reference, respectively**. Figure legends are similar to those in Additional file 3: Figure S2. Click here for file

Additional file 6 **Figure S5: The relation between number of regulatory mouse miRNAs predicted by PITA method and protein evolutionary rate using human (A), chimpanzee (B), cow (C), and chicken (D) as reference, respectively**. Figure legends are similar to those in Additional file 3: Figure S2. Click here for file

Additional file 7 **Table S2: Correlation between 3'UTR length of mRNAs and protein evolutionary rate using various species as references**. Correlation shown in the table is Spearman correlation coefficient. Click here for file
